# On target methods to induce abscopal phenomenon for Off‐Target effects: From happenstance to happenings

**DOI:** 10.1002/cam4.5454

**Published:** 2022-11-21

**Authors:** Blessie Elizabeth Nelson, Jacob J. Adashek, Steven H. Lin, Vivek Subbiah

**Affiliations:** ^1^ Department of Investigational Cancer Therapeutics The University of Texas MD Anderson Cancer Center Houston Texas USA; ^2^ Department of Oncology The Sidney Kimmel Comprehensive Cancer Center, The Johns Hopkins Hospital Baltimore Maryland USA; ^3^ Department of Radiation Oncology The University of Texas MD Anderson Cancer Center Houston Texas USA

**Keywords:** abscopal, cancer, cellular therapy, nanoparticles, radionuclide therapy, Radscopal, systemic therapy, vaccine

## Abstract

Although the “abscopal phenomenon” has been described several decades ago, this phenomenon lately has been obtaining momentous traction with the dawn of immune‐based therapies. There has been increased cross talk among radiation oncologists, oncologists and immunologists and consequently a surge in the number of prospective clinical trials. This must be coupled with translation work from these clinical trials to aid in eventual identification of patients who may benefit. Abscopal effects may be induced by local and systemic methods, conventional radiotherapy, particle radiation, radionucleotide methods, cryoablation and brachytherapy. These approaches have all been reported to be stimulate abscopal effect. Immune induction by immune checkpoint therapy, immune adjuvants, cellular therapy including CAR and NK cell therapies may generate systemic abscopal response. With increasing recognition of this effect, there remains a lot of work to explore the modalities of inducing abscopal responses and ultimate prediction or prognostication on stratifying who may benefit. Ultimately, there is an urgent need for prospective studies and data to tease apart which one of these modalities can be applied to the appropriate candidate, to the appropriate cancer at the appropriate setting. This review seeks to elucidate readers on the different modalities of radiation, systemic therapies and other techniques rarely explored to potentiate the abscopal effect from a mere coincidence to a finite occurrence.

## Graphical abstract

Harnessing the precision power of radiation therapy to cancer sites has been an important part of cancer therapies however, we are becoming more aware that radiation is not only killing cancer cells at the cancer sites but also around distant sites away from the radiation field and hence controlling the spread of cancer all over the body, the effect now known as the abscopal effect. This review seeks to elucidate to readers the different modalities of radiation, systemic therapies, and other techniques rarely explored to potentiate the abscopal effect from a mere coincidence to a finite occurrence.

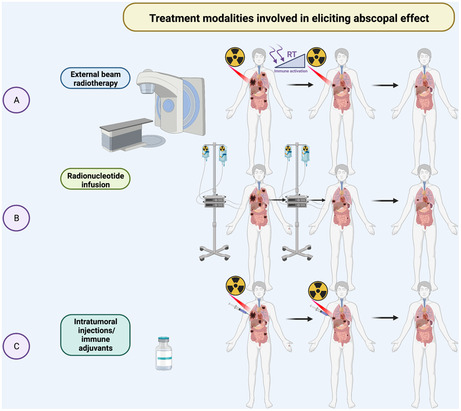

## 1 INTRODUCTION

The abscopal effect has been described for decades as a phenomenon where distant tumors outside of a radiation field shrink in response to treatment that was not directed toward them.^1^ R. H. Mole coined the word first “abscopal” (‘ab’ ‐ away from; ‘scopus’ ‐ target) in 1953 to describe radiation effects “at a distance from the irradiated volume but within the same organism” when he observed this phenomenon in 2 cases of lung and esophageal cancer.^2^, ^3^ This has been thought to be due to inherent stimulation of the immune system from radiotherapy and has caused a great deal of discussion among physicians who treat cancer via a plethora of modalities: radiation oncologists, medical oncologists, immunologists. The increased use of immunotherapeutics in nearly all tumor types has seen an increase in this abscopal effect and led for more investigation into this phenomenon. Efforts into how to identify which patients may benefit from this effect and how to employ this effect continue to be studied and tested in laboratory settings and through prospective clinical trials. Given the renewed interest in this effect in the era of immunotherapy in this review we explore reviewing the evidences of local and systemic therapies that has shown abscopal responses.

### 1.1 Biology of abscopal effect

On a molecular level, radiotherapy causes tumor necrosis which leads to release of damage‐associated molecular patterns (DAMPs) identified by the innate immunity and believed to enhance the immune tumor response.^4^ A key player in the immune tumor response is the natural killer (NK) cells which are activated when DAMPs are presented to them enhancing their antitumor activity. Other various factors such as Type I interferons, CXCL9, CXCL10, and CXCL16 are also increased following radiation therapy and cause further stimulation of the immune system leading to abscopal responses. However, predominantly, neoantigens or tumor associated antigens (TAAs) released during radiation of tumor tissue initiates an immunological cascade of events by dendritic cells who present to CD8+ T cells. These primed cytotoxic T cells initiate tumor specific tumoricidal activity both locally and distally and mediate the abscopal phenomenon.^5^

### 1.2 Methods

Systematic literature search was performed to find published reports, primarily in peer‐reviewed literature, using electronic databases such as MEDLINE via PubMed, Embase and Google Scholar from 1950 to 2021. As this was a literature review, a formal adjudication was not done. Combinations of the keyword “abscopal effect” with any of the following terms were used for the search in the database: “radiation therapy” “metastasis,” “systemic therapy,” “immunotherapy,” “cellular therapy”, “radscopal effects”, “cancer”, “particle therapy” and “oncology.” We also searched the reference lists in the publications that we obtained to find additional relevant publications.

### 1.3 Local techniques to induce abscopal effect

**FIGURE 1:**
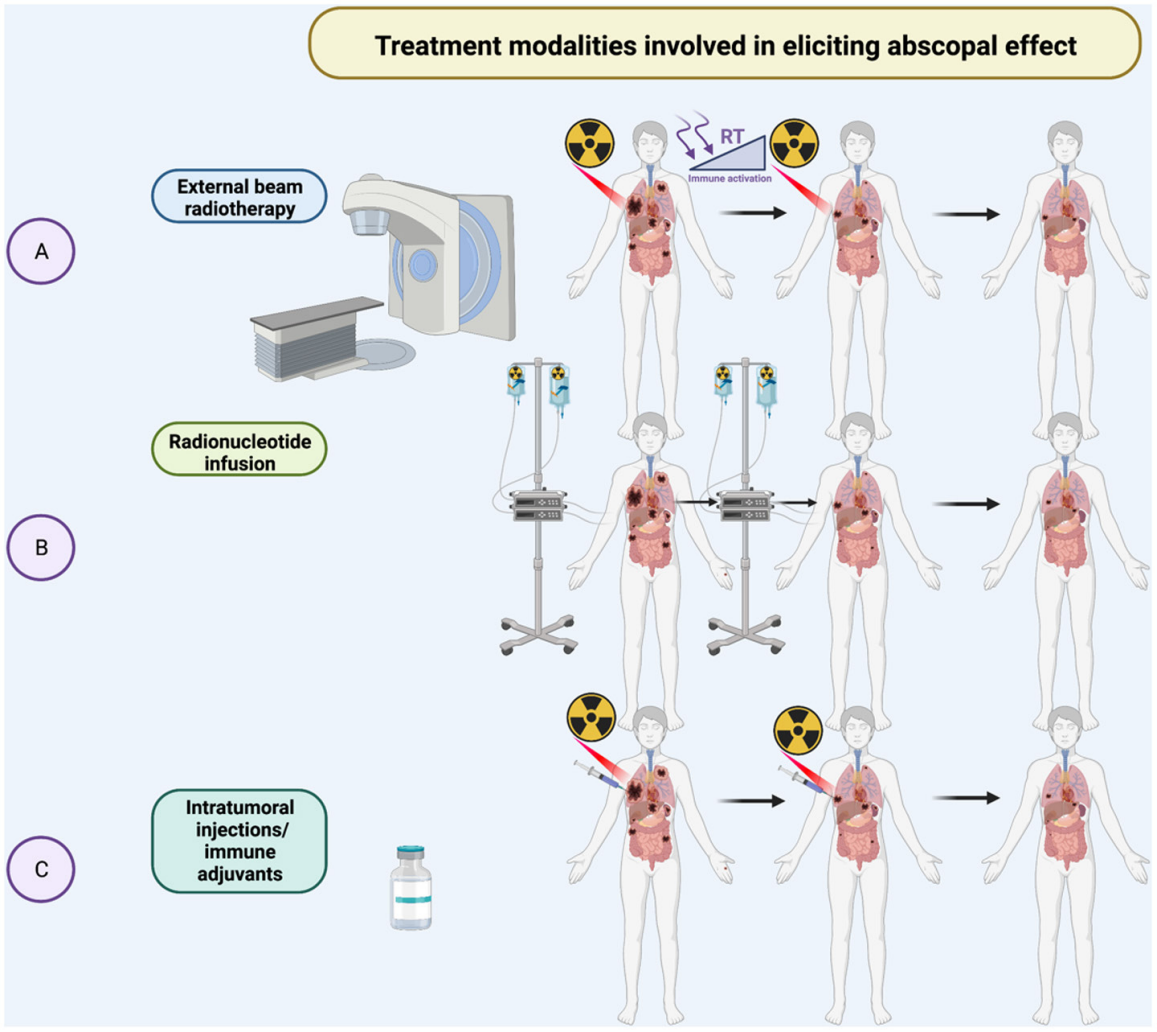
Techniques of abscopal effect. In panel **A**, a patient is receiving external beam radiotherapy to one of their metastatic tumors, over time this targeted radiation leads to immune activation as well as shrinkage of the treated tumor. The treated tumor shows shrinkage and other metastatic untreated sites have regressed likely from activation of the immune system via the abscopal effect. Panel **B** represents a patient receiving an infusion of a radionucleotide therapy for systemic treatment of their advanced cancer. Over time with infusions multiple sites of disease are responding to therapy likely from direct tumor effect of the infusion treatment and immune activation for robust response. Panel **C** is an example of intratumoral injections of a primary tumor and external beam radiotherapy, which may work synergistically to both show shrinkage of other sites of disease through immune activation.

Field size, technique, dose and fractionation matter when combining radiation and systemic therapy to enhance immunogenicity and prevent bystander effect as in Figure 1.^6^, ^7^

### 1.4 Conventional radiation

Conventional fractionation clinically is delivered in doses ranging from1.8 Gy–2 Gy per day. It is well known that lymphocytes are susceptible to RT, hence, consecutive treatment dosage of radiation may exhaust the amount of migrating immune effector cells.

Siva et al reported 10 cases between 1973 and 2011 where abscopal response was noted with usage of conventional radiation. The dosing schedule ranged 12–60.75Gy in 2–27 fractions with median dose of 36 Gy. An impressive durability of abscopal responses was noted at a median of 21 months.^8^

One of the seminal trials by Golden et al^9^ combined conventional radiation with 35Gy/10# with granulocyte‐macrophage colony‐stimulating factor (GM‐CSF) for dendritic cell differentiation and activation in multiple tumor types. An impressive abscopal response of 27% in 11 patients was seen among 41 patients accrued especially in four patients with non‐small‐cell lung cancer, five with breast cancer, and two with thymic cancer. These preclinical and clinical studies continue to show the impact of conventional XRT and its crucial role in reproducing abscopal response in daily clinical practice.

### 1.5 Hypofractionated radiation

A preclinical study examined in‐vivo head and neck SCC patient derived xenografts where anti‐PD‐1 agent was administered with 16Gy/2 fractions (#) high‐dose hypofractionated RT schedule or 20Gy/10# of low‐dose daily fractionated RT schedule. They were able to demonstrate that daily fractionated RT conserved circulating and cytotoxic T cells in the tumor environment, while negatively impacting the immature myeloid cell population but unfortunately, it did not impact Treg population.^10^ Zhang et al showed that abscopal effects were similar in mice with melanoma tumors when hypofractionation radiation regimens comparing 9Gy/3# or 30Gy/10# with similar biological equivalent dose (BED) were delivered.^11^

### 1.6 Stereotactic radiation

Studies show high doses employed in stereotactic radiotherapy would be more effective than conventional doses as the occurrence rate of abscopal effects in preclinical models increases with biological equivalent doses.^8^, ^12^

Seung et al showed that under the influence of interleukin‐2 combined with stereotactic body radiation therapy (SBRT) in metastatic melanoma and renal cell carcinoma patients, 66% ORR was achieved. A high activation of effector cytotoxic T cells were seen in the responders. Patients received 20Gy per fraction for one to three doses of SBRT and the treatment response was dose dependent.^13^

Chicas‐Sett et al performed a systematic review of literature looking at SRS/SBRT and ipilimumab and the incidence of abscopal responses noted. Out of 16 prospective and retrospective studies, abscopal response of 26.5% was seen with improved patient outcomes were found to be a clinically relevant phenomenon. Patients who were administered 3Gy/fraction along ipilimumab with absopal responses experienced improved clinical outcomes.^14^ SRS dosing in the studies varied between 14‐30Gy in 1–10 fractions whereas SBRT dosing schedules varied widely with each study.^14^ SRS/SBRT secondary to employment of high doses lead to exhaustive neoantigen tumor burden and antigen presentation leading to stimulation of cytotoxic T cells and durable cellular mediated immunity and is the most frequently employed RT technique in many studies.^14^

Verbrugge et al showed that high dose SBRT (12Gy/1#) radiotherapy untouched the population of cytotoxic T and natural killer cells crucial to enhancing abscopal response along immunotherapy. In preclinical breast tumor models, radiation enhanced upregulation of effector T cells when biomarker studies were performed.^15^, ^16^ In fact, radiation schedules (30Gy/5# or 24Gy/3#) along with checkpoint inhibitors also showed abscopal response in preclinical models.^17^ So far, little clinical evidence with hyperfractionated regimens (<1.8Gy/#) has been seen which has allowed SBRT with high dose ablation technique to be an attractive technique in eliciting abscopal response.

### 1.7 Particle therapy and radionuclide therapy

Particle therapy, such as protons and carbon ions have a favorable dose distribution and varying dose deposition profiles to target dose delivery at the tumor site and minimize surrounding toxicities. The properties of a photon ray when contrasted with the physical properties of proton ray vary considerably which lead to contrasting dose distributions on application. Due to the focused nature of particle therapy leading to precise targeted dose delivery, bystander radiation effects are not seen. This spares the circulating lymphocytes and peritumoral sparing essential for abscopal response. Carbon ion radiotherapy (CIRT) have a similar ionization distribution to the DNA molecule and hence, this allows full dose delivery in the target volume and cause targeted DNA damage in the tumor without lateral scattering. However, proton therapy has varying radiobiological effectiveness (RBE) along the pathway but majority of the ionization events occur at the end of the beam trajectory.^18^

Studies have showed that particle therapy immunomodulate the tumor and the microenvironment similar to photon therapy. High linear energy transfer helium ion therapy and carbon ion radiotherapy induce a high level of immunogenic cell death proteins such as calreticulin, HSP‐70, DNA and others which stimulates the cGAS/STING pathway for radiogenic tumor death.^19^ Takahashi et al showed in murine osteosarcoma models that when combining CIRT targeting PDL‐1 and CTLA‐4 receptors, an abscopal response rate of 64% was noted compared to 20% in the CIRT group alone.^20^

Brenneman et al reported an intriguing case of metastatic retroperitoneal sarcoma who received upfront proton therapy for local symptomatic control and was noted to experience abscopal response in the out of field distant tumor burden. Patient received 50Gy/25 fractions to the retroperitoneal mass and regional nodal regions and was noted to have near complete response in the primary tumor. It is notable that there was progression initially at the out of field sites when receiving radiation but then after 5 months after radiation completion and in the setting of active surveillance.^21^ Due to the target nature of proton radiation of the primary RPS, no compromise in the T cell population in the tumor environment and blood and nodal regions were trended with radiation and after when measured. Preclinical work from Gamerio et al show that tumor cell lines exposed to proton therapy and photon therapy had similar immunogenic modulation profile and T cell mediated cytotoxicity.^22^

Abscopal responses have also been reported with carbon ion radiation therapy (CIRT). Zhang et al revealed how a patient with metastatic thymic carcinoma received 60Gy in 12 fractions with a Relative Biological Effectiveness (RBE) of 3. Patient was noted to have cancer control in the irradiated lesion and in the out of field lesions and went into remission soon after.^23^ Carbon ions have a higher RBE due to the bragg's peak with improved dose deposition that is specific for at the exit of the beam and non‐dependence on the oxygen content of the lesion which makes it an attractive target in eradicating radioresistant, hypoxic tumors. All these factors make particle therapy attractive to harness the abscopal response potential in clinical practice.

The use of radioactive agents, such as yttrium‐90, in combination of immune checkpoint blockade has also shown to have benefit in a patient with renal cell carcinoma.^24^ This case was an example of radioactive particle therapy in a liver metastasis while the patient was also receiving systemic nivolumab had shrinkage in all other metastatic sites.^24^

Bellia et al reported on seeing abscopal effect in cutaneous SCC when treated with diffusing alpha emitters radiation technique. Patient has a complete regression of in field and out of radiation field lesions with durable response lasting more than a year.^25^

### 1.8 Unconventional techniques

In clinical practice, we may find that some tumors have a large irradiation field, and complete ionization radiation of these massive lesions could cause increased organ at risk toxicities. A few unconventional approaches have been devised for ablative cytotoxic radiation doses to the lesion while conserving the surrounding immunogenic tumor microenvironment and normal tissues.

GRID (Spatially fractionated radiation therapy) block technique using high dose variable radiation spatially was established to administer radiation via Multi Leaf Collimator (MLC) in bulky lesions.^26^ A similar technique known as Lattice radiation also administers ablative variable doses of RT to large tumors and helps with preserving surrounding organs at risk.^27^ SBRT‐PATHY (stereotactic body radiation therapy‐based partial tumor irradiation targeting hypoxic segment of bulky tumors) is another technique that was developed by Tubin et al to harness hypoxia in bulky tumors where stereotactic body radiation therapy is administered to partial tumor mass irradiation technique.^19^, ^27^ FLASH‐RT (FLASH Radiotherapy_ which utilizes novel radiotherapy technology with single ultra‐high dose‐rate (≥40 Gy/s) radiotherapy and microbeam radiation therapy are lesser known techniques that are being investigated for abscopal response as well).^19^, ^28^ Out of these techniques, the SBRT‐PATHY has shined to hold relevant promise in inducing abscopal response as it takes into account radiation‐induced bystander effect, manipulating tumor hypoxia, protecting the peritumoral environment and partial irradiation. Tubin et al showed in their preclinical work with inducing hypoxia in A549 and H460 lung cancer cell lines and then exposing them to irradiation, there was significant bystander effect and tumor growth delay.^29^ This led to a phase 2 trial where SBRT‐PATHY technique was used in sixty patients with bulky advanced lung carcinomas. And 3 arms were compared using SBRT‐PATHY, chemotherapy, and palliative radiotherapy. Highest abscopal responses were seen in the SBRT‐PATHY arm at 95%. At 1‐year, the SBRT‐PATHY arm experienced the highest median overall survival (OS) at 75% and highest tumor control at 95% compared to 60% OS in chemotherapy arm and 20% OS in palliative radiotherapy arms with clinical significance.^30^

There is much need in translational work in understanding if differential modalities elicit abscopal responses in varying fashion as currently this is lacking in literature. Irradiating one single site is likely not enough to produce tumor‐associated antigens resulting in uncertain and unreproducible effects in abscopal studies currently. Therefore, an alternative tactic could be to irradiate as many lesions as possible in combination studies to increase antigen presentation and neoantigen tumor burden to harness the T cell activation.^31^ For eliciting cytotoxic tumoricidal killing, an ablative dose of ionizing radiation should initiate tumor cell death cascade leading to tumor antigen production. This in turn released immune cell death signatures and proteins or adjuvants to promote and differentiate dendritic cells and macrophages to improve effective antigen representation. This leads to activate of effector CD4 and CD8 + cytotoxic T cells for effective tumor response. Extensive preclinical and clinical investigations regarding establishing a standardized radiation schedule, the type of modality and fractionation to maximize immunogenic cell death is currently underway. Other important factors include coordinating sequence of immunotherapy and radiotherapy, understanding the synergy of myriad of systemic agents that could complement the ionizing effects of radiation and overcoming the inhibitory state of the tumor microenvironment involved in the abscopal response interplay. These variables which need further investigative studies will be crucial to evaluation, quantification and reproducibility of the abscopal effects in clinical practice (Table 1).^32^

**TABLE 1:** Multimodality clinical trials demonstrating evidence of abscopal response in literature

Modality	Tumor type (*n*=)	Systemic agent	Median os (months)	Abscopal response %
Conventional 35Gy/10#	Non‐small‐cell lung cancer (*n* = 13)	Chemotherapy+GM‐CSF	20.98 m	27%^9^
	Breast cancer (*n* = 11)			
	Thymic cancer (*n* = 2)			
SBRT 40‐50Gy/5# and hypo fractionated brachytherapy (30Gy/1#)	Non‐small‐cell lung cancer (*n* = 23)	Nivolumab	NR	45.2%^37^
Conventional 50Gy/25# and HDR (24Gy/3#) +/− Electro hypothermia	Cervical Cancer (*n* = 108)	Cisplatin	Not assessed	24.1%^90^
SBRT 50‐60Gy/4–10#	Multiple Tumor Types (*n* = 106)	Ipilimumab	NR	26%^45^
Proton Therapy 25Gy/5#	Head and neck squamous cell carcinoma (*n* = 31)	Durvalumab and Tremelimumab	6.4 m	27.3%^91^
Conventional or SBRT	Non‐small cell lung cancer (*n* = 17) Melanoma (*n* = 5)	Immunotherapy	Not assessed	11%^92^
Electroporation six pulses, 1500 V/cm) on days 1, 5, and 8 every 90 days	Melanoma (*n* = 28)	Intratumorally with plasmid encoding IL‐12 (tavokinogene telseplasmid	NR	29.2%^93^
Conventional or SBRT 24Gy/3#; 50Gy/4#; 45Gy/15#	Lung Cancer (*n* = 148)	Pembrolizumab	19.2 m	41.7%^94^
Low Dose Radiation (4Gy/2#)	Follicular lymphoma (*n* = 51)	Intratumoral G100 (TLR4 agonist) +/− Pembrolizumab	NR	16.2%^95^
SBRT 30Gy/3#	Renal Cell Carcinoma (*n* = 69)	Nivolumab	20 m	12%^96^
SBRT 6 or 8 Gy (2–3#)	Melanoma (*n* = 22)	Ipilimumab	10.7 m	22.7%^97^
SBRT 48Gy/3–5#	Non–Small Cell Lung Cancer (*n* = 34)	PD‐1/PD‐L1 inhibitor +/− Chemotherapy	15 m	53%^98^
Conventional or SBRT 27‐70Gy/3–25#	Colorectal Cancer (*n* = 24)	Durvalumab and Tremelimumab	11.4 m	8.3%^99^
SBRT 24Gy/3#	Colorectal Cancer (*n* = 40)	Ipilimumab+Nivolumab	7.1 m	10%^100^
	Pancreatic Cancer (*n* = 25)		4.2 m	12%

## 2 ABSCOPAL EFFECTS BY OTHER MODALITIES

### 2.1 Cryoablation

Another patient with metastatic SCC of the hard palate with recurrence in the neopharynx and left parotid bed mass underwent emergent salvage cryoablation to avert aerodigestive tract obstruction. At 8 months post cryoablation both in the field of radiation and out of field with near‐total resolution of the 4.5 × 4‐cm left parotid bed tumor recurrence consistent with abscopal response via cryoablation.^33^

### 2.2 Brachytherapy

Brachytherapy by itself is not commonly used as a single adjunct to elicit abscopal response. However, Suzuki et al report a case of metastatic renal cell carcinoma who received high‐dose‐rate interstitial brachytherapy along with immunotherapy to the left iliac crest tumor (9.5 × 8.8 cm). At 3 months after brachytherapy, the primary tumor responded to RT along with the regional left internal iliac node. Along with this, spinal metastases in L4 completely disappeared indicative of abscopal response.^34^

### 2.3 Photosensitizer therapy

Xie et al proved a novel preclinical approach in colorectal cancer cell lines and murine models with, IR700DX‐6 T which is a translocator protein targeted photosensitizer which caused neoantigen production leading to tumor growth suppression in metastatic sites. Stimulation of activated T cells and DCs with inhibition of regulatory T cells were seen in both administered and non‐administered lesions. Translating this approach to clinical studies holds much promise for induction of abscopal responses.^35^

## 3 ABSCOPAL EFFECTS IN SYSTEMIC THERAPY

Prior literature has affirmed the role of RT in mediating immunogenic abscopal effect, albeit being rare, with only 46 reported cases presently.^36^ As a single modality, radiation is able to induce abscopal effects limitingly due to lack of persistent and distant T cell activation, upregulation of MDSCs, regulatory T cells, and checkpoint ligands.^37^ This necessitate combining this promising modality with systemic therapy to augment the tumoricidal effects and enhance cytotoxic T cell activity.

Searching for the ideal combination of radiation and systemic therapy, staggering in the most effective fashion along with the optimal dose of ionizing radiation may need to be tailored to the appropriate tumor and its proclivity to systemic agents which need to be tested in prospective studies.

### 3.1 Immunotherapy

Checkpoint inhibition of CTLA‐4 and PD‐1 ligands has revolutionized the world of immunotherapy in mitigating T cell inhibition, but this is highly dependent on the vital interface with CD28 receptor on differentiated antigen presenting cells. Radiation potentiates the process by ramping up neoantigen tumor burden converting “cold” tumors to “hot” tumors.^38^ Review of literature show abscopal responses ranging from 20%–50%.^39^ Many prospective trials have looked at clinical outcomes and abscopal responses with use of immunotherapy and radiation with promising results requiring further validation.^40^, ^41^ Anti‐PD‐1/PDL‐1/CTLA4 administration after irradiation of primary or metastatic lesions causes modulation of the T cell repertoire, development of effector T cells with recalibration of aberrant cytotoxic T cells to stimulate abscopal responses. In this preclinical study, sequencing of PD1 antibody before radiation increased the effector T cell population in the radiation tumor leading to immunogenic cell death and subsequently extending responses by suspension of differentiation of effector T cells to sustain intratumoral and distant responses.^42^ Other studies suggest that abscopal responses were more likely when radiation was combined concurrently with PD1 antibody while with CTLA‐4 antibody, this was seen when radiation was sequentially administered after.^43^

Interestingly, Mihaylov et al were able to elicit abscopal response in murine models where abscopal response rate of 26% were noted when treated with 24Gy/3# followed by PD‐1 antibody therapy which was validated by CT and MRI radiomics imaging and lower pretreatment neutrophil to lymphocyte ratios.^44^ In clinical practice, one phase 2 trial looked at sequencing combinatory or sequential SBRT with optimal delivery of ipilimumab in tumors with liver or lung spread. Out of 106 patients, clinical benefit rate (CBR) in the distant out of field lesions were 26% in both arms while it was higher (28% vs 20%) when ipilimumab was staggered after RT in contrast to simultaneous administration. The greatest benefit was seen for lung lesions at 42% CBR.^45^ On the other hand, a phase II study found that nivolumab along with radiation (stereotactic) (8Gy/3#) concurrently in 20 patients with advanced malignant melanoma demonstrated an ORR in the out of field lesions at 45%.^46^ So it yet remains to find a consensus on the timing of immunotherapy administration for eliciting an effective and reproducible abscopal response. It should be noted that ipilimumab (every 3 weeks for 4 doses) was given with radiotherapy begun during the first dose (concurrent) or 1 week after the second dose (sequential) and delivered as 50 Gy in 4 fractions or 60 Gy in 10 fractions to metastatic liver or lung lesions and hence the abscopal responses derived secondary to the combination mutually.

Abscopal responses can also be seen after developing immunotherapy resistance as seen in the retrospective study by Grimaldi et al.^47^ They found 21 candidates who progressed after receiving ipilimumab q3weekly and had been administered palliative radiation for symptom control. Interestingly, 11 patients (more than 50% in the retrospective cohort) demonstrated abscopal responses in the out of field lesions lasting between one to 4 months. These patients also had a median OS greater than 1 year compared to those did not develop abscopal responses. Notably, the median time from the start of ipilimumab treatment to RT was 5 mo (range: 3.4–8) and although its not clear if only RT was instrumental in eliciting these abscopal responses, authors note that the incidence of delayed responses was much higher than would be expected with ipilimumab treatment alone.

TTI‐621 is another promising agent in this space being explored for abscopal responses which is a fusion protein linked to CD47 pocket of the signal‐regulatory protein alpha and the immunoglobulin IgG1. IgG1 blocks its interaction with MDSCs and activates the “eat‐me” signal in tumor environment. Intralesional delivery of TTI‐621 in 66 patients with mycosis fungoides or sézary syndrome show abscopal response in 80% of patients.^48^

### 3.2 Targeted therapy

Many human tumors have upregulation of the Transforming growth factor β (TGF‐β) expression that promotes a therapy‐resistant phenotype which in turn creates epithelium plasticity and mesenchymal differentiation leading to an immunosuppressive tumor environment. TGFβ negatively interacts with the adaptive and innate immunity downregulating effector T cells and NK cells leading to an immune resistant tumor landscape.^49^ Twenty‐three patients with metastatic breast cancer were examined in randomization in a trial where fresolimumab which is a monoclonal antibody targeting human TGF‐β1‐3 was combined with radiation. Three patients demonstrated stable response. Clinical significance was noted with synergy with ablative doses of radiation (22.5Gy/3#) and increased fresolimumab dosing correlating with an improved OS (*p* = 0.039) with mechanistic demonstration of increase in activation of T cells although no abscopal response was noted.^50^

Hotta et al report a curious case of metastatic lung cancer with brain metastases who received WBRT at 30Gy/10# while on osimertinib 80 mg qday with 35% shrinkage in her primary left upper lobe lung lesion, possibly revealing abscopal response with EGFR inhibitor, albeit abscopal response lasting for 45 days.^51^

Cerbone et al reported a case of metastatic RCC with lung metastases who had abscopal response after XRT to the subcarinal lymph nodes (30Gy/10#) with ongoing response with PR at the irradiated site and treatment response at bilateral lung metastatic sites. It is notable that patient had stopped pazopanib 6 months prior to XRT but the authors hypothesize that VEGFR antagonists are vital in immunomodulation with possible persistence of cell memory which could have played a crucial role in creating a robust abscopal response.^52^

Dengina et al revealed in their prospective study where one patient had abscopal response (6%) out of 17 patients when on systemic therapy with everolimus and receiving SBRT to the distant lung lesion. After 6 months, abscopal response persisted in the primary and distant lung lesions.^53^

Morales‐Orue et al shed light on the potential for nanoparticles to potentiate abscopal responses as a targeted modality by being able to overcome drug resistance through physical and molecular barriers and by being able to capture antigens for presentation effectively.^54^ Min et al showed that antigen‐capturing nanoparticles (AC‐NPs) delivered cancer‐related neoantigens to APCs effectively and improved the efficacy of PD1 antibody therapy in melanoma and orthotopic breast cancer tumor models when combined with XRT (8Gy/3#). Complete abscopal response was noted to be at 20% in the AC‐NPs arm while none was seen in the control arm. Translational work showed that exposure to these nano particles stimulate differentiation of effector T cells while decreasing the regulatory T cell population.^55^ Exploring this space continues to hold promising potential for abscopal effects with XRT.

Bintrafusp alfa, a bifunctional fusion protein leveling TGF‐β and PD‐L1 blockade overcomes an immunosuppressive tumor environment in murine models by increasing TIL expression and infiltration, stimulation of interferon genes (STING), decreasing PMN‐MDSCs and leveraging TGF‐β trap to PD‐L1 blockade. This agent was also shown to be able to elicit abscopal responses in lung lesions distantly with durable responses.^56^

### 3.3 Immunoadjuvants

Immunoadjvants are agents added to or used in conjugation with a vaccine antigen to augment or potentiate and possibly target the specific immune response to the antigen. Because radiotherapy essentially converts cancer lesions into an “in‐situ” vaccine, synergizing with ideal immunoadjuvants could potentiate immunogenic primary and abscopal responses of the tumor and the microenvironment.

Many preclinical studies have employed anti‐CD40, LFT3‐L, ECI301, IL2, 9H10, DC as immunoadjuvants to boost abscopal responses in tumor models.^57^

Anti‐CD40 antibody have been tested to enhance activation of APCs. Yasmin‐Karim et al used Intra‐tumoral Smart Radiotherapy Biomaterials (SRB) to administer CD‐40 targeted antibody in murine models along with 5 Gy of RT in the subcutaneous tumors. Results indicated that a significant reduction in distant tumor volume (96–98%) occurred with increased survival with increased recruitment of effector T cells leading the authors to conclude anti‐CD40 antibody to be an effective immunoadjuvant with radiation to potentiate abscopal responses.^58^

Granulocyte macrophage colony‐stimulating factor (GM‐CSF) is a known potent immunoadjuvant to expand and prime the dendritic cells and antigen presenting cells. Newcomb et al showed in murine intracranial glioma models, peripheral vaccination with GM‐CSF as monotherapy did not affect murine outcomes. Nevertheless, when it was combined with ionizing radiotherapy, more than 80% of the murine models experienced better outcomes.^59^ This translated to seminal clinical findings in the prospective study by Golden et al where patients with multiple tumor types received GM‐CSF administered for 2 weeks with radiation and investigator's choice of chemotherapy where an impressive 27% abscopal response rate was noted. This also translated to clinically significant improvement in OS with abscopal responder patients compared to their counterparts where the median overall survival was 20·98 months for abscopal responders where as it was 8·33 months for those who did not develop abscopal responses (HR 2·06).^9^

In a basket study with advanced solid tumors, 15 trial candidates were administered cyclophosphamide followed by intratumoral polyinosinic‐polycytidylic acid‐poly‐l‐lysine carboxymethylcellulose (poly‐ICLC) which is a TLR‐3 agonist and dendritic cell injection x 4 days. 50% of patients had RT with 17% (*n* = 1) abscopal response rate although it should be noted that only one patient experienced this effect.^60^ This agent was also used in a phase II single arm study with recurrent anaplastic glioma in which poly‐ICLC along conventional radiation to the recurrent brain tumor in 60Gy in 30 fractions. Patients went on to receive poly‐ICLC maintenance therapy for 12 months, or until disease progression was noted. And 30 patients had a median OS of 69% at 12 months, although abscopal responses were not seen.^61^

Sadahiro et al looked at addition of polysaccharide krestin (PSK) known to be mushroom based protein‐bound polysaccharide, concomitantly with neoadjuvant chemoirradiation in locally advanced rectal cancer along with S1. PSK is derived from Trametes versicolor which activates the expansion of cytotoxic T cells and APCs via toll like receptor −2 for immunological stimulation. Mechanistic studies revealed concordant rise in CD8+ and CD4 T cells and NK cells in the tumor microenvironment.^62^

Ngwa et al advocate rationale to employ subcutaneous autografts as immunoadjuvants to control the priming process in generating of abscopal responses. Identifying an autologous pre‐existing subcutaneous tumor for irradiation circumvents issues with radiation planning, dealing with organs at risk, improved use of higher LET radiation to induce tumor immunogenicity and peripheral APC presentation for targeting neoantigens. This also allow systemic immunoadjuvants to be used intra‐tumorally to synergistically increase abscopal response rates.^57^

Herrscher et al reported in 2021 about a case developing abscopal response in metastatic BRAF V600E mutated malignant melanoma who had stopped targeted BRAF/MEK therapy 3 months prior when she received palliative RT of 20Gy/5# to bulky cervical adenopathy. She developed COVID19 infection within 1 month of completion of RT and restaging scans showed abscopal response with 20–25% shrinkage of the non‐irradiated distant metastases in the peritoneum and lymph nodes. Although the authors conclude that the likely abscopal response was secondary to severe acute respiratory syndrome coronavirus 2 (SARS‐CoV‐2), the combinatory effects of radiation which can last many months post treatment could have mediated a crucial role in the abscopal phenomenon.^63^

### 3.4 Cellular therapy

While immense preclinical and clinical evidence is seen with radiation and immunotherapy, to date there is dearth of translational validity to assess the combination of radiation and CAR T‐cell therapy. However, with increasing use of CAR‐T cell therapy, there are reports of abscopal responses with synergistic use highlighting its potential in this space. A preclinical study found that focal irradiation allowed specific tumor antigenic targeting of NKG2D‐based CAR T cells against glioma models leading to effective destruction of tumor cells by CAR‐T infiltration.^64^ Smith et al report a case of refractory myeloma who received B‐cell maturation antigen targeted CAR T‐cell therapy followed (on Day 6) by palliative radiotherapy to the brain and spine (20Gy/5#). Notably, after RT, she developed cytokine release syndrome like picture and hematological evaluation revealed more than 30% T‐cell repertoire propagation of CAR‐T cells at 5 weeks compared to baseline after CAR‐T infusion. Restaging after cellular therapy showed complete response both in the in‐field and out of field lesions consistent with abscopal response with durability up to 9 months.^65^ Also, intrapleural delivery of cancer‐antigen mesothelin targeted CAR‐T cells were administered to a murine model with prior focal thoracic XRT which elicited a systemic abscopal response as well. This is yet to see potential in the clinical space at this time.^66^

One of the earliest preclinical studies demonstrated synergy when IL‐15 which facilitates release of effector T cells and NK cells, and ionizing radiation potentiated a cellular mediated immune response in the tumor and systemic circulation. Addition of DTA‐1 which is a glucocorticoid‐induced tumor necrosis factor receptor–related protein agonist sustained this response longer.^67^ However, a review by Chen et al reveal preclinical studies have successfully harnessed NK functions with low dose XRT while with high dose XRT, activation of NK cells occur when pre‐emptively treated with IL‐2. These preclinical findings need to be translated to clinical practice with radiation to confirm and explore the potential of NK therapy in eliciting abscopal response with XRT.^68^

Sato et al demonstrate the ability of adoptive T cell therapy (ATC) in combination with irradiation in a metastatic gastric cancer patient with peritoneal metastases who received ATC and dendritic cells via IV and local injection Q2weekly. IMRT with a dose prescription of 48 Gy in 24 fractions was used to irradiate the primary tumor along with concurrent ATC therapy. Abscopal responses was noted in the peritoneal lesions along with primary tumor shrinkage with response lasting 5 months.^69^

Synergy between cellular therapy and radiation may occur secondary to the cytokines secreted by CAR T cells, increasing the likelihood of endogenous T cells mounting an abscopal‐like response leading to signaling through the T Cell Repertoire (TCR) of CAR‐T cells which may enhance clonal CAR T‐cell expansion.^65^ Prior translational work have demonstrated that RT may enhance effector functions and migration of CAR T cells.^70^ This space continues to be an evolving field which holds promise in combination with irradiation. Further studies are warranted to establish its abscopal potential in clinical practice.

### 3.5 Chemotherapy

Review of literature indicate some chemotherapeutic agents such as Mitoxantrone, Adriamycin, Etoposide, and Oxaliplatin induce immunogenic cell death which when used with RT as an immunogenic hub potentiates abscopal responses.^71^ Tumoricidal killing was first harnessed by cytotoxic chemotherapy which let to efficient priming of the adaptive immune system improving responses.^72^ Similarly, in response to anthracyclines, cancer cells form molecular bonds with calreticulin and glucose‐regulated protein, 58‐kD (GRP58) on the membrane with upregulation. This allows trafficking of the damaged tumor cells by the APCs. Tumors that undergo alternative forms of cell death with mitomycin‐C and topoisomerase inhibitors can stimulate immunogenicity by bonding of calreticulin to the membrane.^73^ Apetoh et al showed that dendritic cells stimulate TLR‐4 transcription which controls the adaptive immunity for a profound cytotoxic response mediated through the T and NK cells. These responses persist due to the high amount of neoantigen burden and ICD proteins.^74^ Although strong preclinical studies indicate the potential of cytotoxic chemotherapy, no particular chemotherapy has been strongly associated with eliciting abscopal responses consistently.

### 3.6 Radionuclide therapy

Radionuclide therapy has been expanding rapidly in the oncology field secondary to innovative radioactive novel molecules being developed for specific targets. The mechanism of radionuclide induced abscopal effect, as in the case of external radiotherapy is modulated immunologically where local tumor cell death occurs by apoptosis and necrosis leading to release of DAMPs and tumor antigens, immunogenic cell death proteins like calreticulin, ATP and HSP‐70, chemokines and cytokines. Tumor cells affect upregulation of cell adhesion molecules, TNF family ligands and major histocompatibility complex class I and II which in turn allow circulating DCs to transfer the TAAs to nodal regions and present to CD4 and CD8 T lymphocytes causing their activation via interaction of T‐cell receptors with peptide/MHC complexes on APCs.^75^ Radiation‐induced bystander effect, abscopal effects and crossfire effect are effective treatment strategies with exposure to ionizing radiation. The crossfire effect predominates in bulky lesions and especially with β‐emitter isotopes who travel a longer trajectory leading to beam scatter between healthy and non‐healthy tissue. Hence defining this approach by using a radioisotope with shorter beam pathway like an alpha‐emitting particle could potentially target tumor volume in a focused manner. Alpha emission particles have a similar ionizing length for targeted helical tumor DNA and chromosomal damage, indiscriminate tumor cell damage even in hypoxic tumors. This holds the key in overcoming hypoxia‐related resistance by employing beta or alpha radiation. Hence as with other modalities radionuclide therapy holds promise in eliciting high amount of neoantigen tumor burden and inducing immunological responses. Alpha‐emitting particles have unique properties with a high LET and shorted beam pathway within the tissue leading to less dose scatter, high intensity of energy deposition at the specified target. It is capable of affecting response regardless of the dosing or oxygen levels or cell state than gamma and x‐rays. Alpha‐emitting particle concept has been incorporated in Edotreotide for well and moderately differentiated somatostatin expressing neuroendocrine cancers and as a prostate‐specific membrane antigen ligand for prostate cancer. In a preclinical study in murine models, radiation with Bismuth‐213 antibody delivered abscopal responses at metastatic regions mediated by the adaptive immunity that was durable with release of DAMPs and HMGB1 to trigger activation of dendritic cells. This study shows the abscopal response potential of alpha irradiation which can stimulate adaptive immunity and release tumor specific antigens for cytotoxic tumoricidal responses.^76^ Czernin et al combined 225Ac‐PSMA617 and an anti‐PD‐1 antibody in mice models and found that the synergy improved tumor control (33.5d vs 25d) with improved survival and no notable toxicities. Although mechanistic studies were not conducted, the authors hypothesize radionuclide therapy synergistically harnesses the cytotoxic T cell system to induce effective local responses akin to RT.^77^

Ghodadra et al demonstrated abscopal response in a patient with metastatic lung cancer after radioembolization with Yttrium‐90 (β‐emitter) of the hepatic metastases. Y90 modality was employed with 1.2 gigabecquerel aimed to target right hepatic lobar lesions caused not only regression of treated metastases, but also disappearance of the non‐targeted, lesions in the left lobe.^78^ Ricci et al showed a case of metastatic castration resistant prostate cancer who received Radium‐223 dichloride therapy and had a complete response and reversal of castration status consistent with abscopal response at distant soft tissue sites although Radium‐223 is mostly bone avid in distributive nature.^79^ This was also confirmed with 223Ra‐Dichloride with abscopal soft tissue response was noted with 2 metastatic CRPC patients.^80^

The radiobiology of targeted radionuclide therapy needs to be investigated to gain the knowledge about its potential for bystander and abscopal effect, which could harness the concept of radionuclide therapy in augmenting abscopal responses with systemic therapy. The challenges one may encounter includes the determination of appropriate dose and dose‐rate effects for different type radionuclides and for particular types of tumors to selectively achieve detrimental effects toward tumor cells while preserve organs at risk and quantify agents that may have more predilection to elicit abscopal response.

### 3.7 Vaccine therapy

Cancers are usually heterogenous in nature due to the diverse antigen presentation on the extracellular membranes. Hence, targeting a specific antigen for vaccine therapy may not be successful. But Newman et al were able to harness the power of cancer vaccines by recalibrating the TME to increase immunological cytolytic effects. The authors used adjuvant free influenza vaccine that was administered intratumorally to transform non‐immunogenic tumors to immunogenic ones and subsequently generate effector T cell‐mediated immune responses and overcome immune inhibition.^81^ This could serve as a fundamental basis for many approaches to elicit a profound abscopal response in less immunogenic tumor histologies.

Other modalities such as cryoablation; electrothermal delivery radiation; immunotherapy were also shown to induce effective immune response.^82^, ^83^ Notably, immunotherapy, RT and cytotoxic therapies were proven modalities with mechanistic evidence to elicit immunogenetic cell death proteins. The delivery and presentation of these released antigens are still limited by immune tolerance and factors involved in the tumor microenvironments. Chen et al have established preclinical studies in murine models with 93‐O17S‐F is a lipidoidal‐based nanoparticles that potentiates in‐situ vaccine strategy by releasing tumor antigens TAAs to stimulate the cGAS‐STING pathway by mediating through interferon exposed to prior administration with doxorubicin. 93‐O17S‐F accelerates neo‐antigen procural through Van Der Waals interaction. By targeting the release of cGAMP which is a STING pathway stimulator to lesion, it could turn the immunosuppressive environment and become a potential and viable strategy to synergize with radiotherapy to induce effective abscopal responses without discrimination of neoantigen capture and delivery.^84^

## 4 RADSCOPAL ™ EFFECTS

In general, doses less than 0.5 Gy cause minimal induction of cell death. They were able to show in the human peripheral blood on exposure to ionizing radiation, natural killer cells were innately fragile compare to B or T cells while myeloid derived lineages were innately more radioresistant in nature.^85^ Cells stressed by low‐dose irradiation create an highly cytokine driven surrounding swimming with effector CD4 and CD8+ T cells, ICD proteins, neoantigens, various TNF and IL factors mediating which cause secondary cytolytic tumor killing. In preclinical studies on cancer cell lines, administration of low dosing of RT induces changes in adjacent tumor environment akin similar to ablative doses targeting primary tumors.^86^

In the Radscopal ™ concept, ablative doses of radiation is administered to primary tumors to for tumoricidal death, tumor antigen production and presentation to APCs for priming of the adaptive immunity. Combining this with low dose radiation administered to other metastatic lesions modulates the stromal and microenvironment through suppression of TGF‐ß and enhancing penetration and differentiation of cytotoxic T cells. Addition of checkpoint inhibitors augments the effects of low dose XRT by reprogramming M1 macrophage differentiation and increasing natural killer cell penetration.^87^

In a phase 2 prospective trial (NCT02710253) in multiple metastatic tumor types where HD‐RT (20‐70Gy total; 3–12.5Gy/fraction) and LD‐RT (LD‐RT comprised of <2Gy/fraction up to 10Gy total) was combined with immunotherapy in 74 patients. The 4‐month overall disease control rate was higher in the combination XRT arm (47%) compared to the HD‐RT arm (37%) although abscopal responses in the non‐irradiated lesions was lower (23%) compared to LD‐RT treated lesions (53%).^88^

The need for combination of both level of doses is required to elicit an abscopal response where high dose XRT primes T cells to recognize neoantigens while low dose XRT reprogrammed the immunosuppressive tumor environment to enable these immunogenic cytotoxic immune cells for tumor penetration. Thus high‐dose radiation in the form of SBRT (6‐12Gy/#) and low‐dose radiation (7.5Gy/5#) have been showed have diverse immunomodulatory effects on harnessing the adaptive and innate immunity for tumoricidal immunogenic killing. Some challenges with this technique include a non‐standardized approach to personalize radiation planning, addressing the organs at risk when multi‐site radiation is targeted, increased patient treatment time and workflow limitations.^89^ The hypofractionated technique, employment of extreme framework of doses and increasing the number of sites for irradiation in theory would allow the probability for abscopal responses to exponentialize.

## 5 SUMMARY AND CONCLUSION

Although abscopal effect has been described several decades ago, this phenomenon has only recently gained traction to be exploited prospectively. With increasing recognition of this effect, there remains a laborious undertaking to explore the modalities of inducing and sustaining a consistent abscopal response across the therapeutic spectrum and develop innovative biomarkers for appropriate patient and tumor selection and prognosticate who benefits from these modalities. Ultimately, we need more prospective studies and data to tease apart which one of these modalities can be applied to the appropriate candidate, to the appropriate cancer at the appropriate setting.

## AUTHOR CONTRIBUTIONS

**Blessie Elizabeth Nelson:** Conceptualization (lead); data curation (lead); formal analysis (lead); investigation (lead); methodology (lead); project administration (lead); resources (lead); software (lead); supervision (lead); validation (lead); visualization (supporting); writing – original draft (lead); writing – review and editing (lead). **Jacob J Adashek:** Visualization (lead); writing – review and editing (equal). **Steven Lin:** Writing – review and editing (supporting). **Vivek Subbiah:** Conceptualization (lead); supervision (lead); validation (lead); visualization (supporting); writing – review and editing (lead).

## FUNDING INFORMATION

None to declare.

## CONFLICT OF INTEREST

**BEN and SHL** report no disclosures. **JJA** serves on the advisory board of CureMatch, Inc. **VS** reports grants from Eli Lilly/LOXO Oncology, Blueprint Medicines Corporation, Turning Point Therapeutics, Boston Pharmaceuticals; and grants from Helsinn Pharmaceuticals; in addition, VS reports a grant and advisory board/consultant position with Eli Lilly/Loxo Oncology during the conduct of the study; research grants from Roche/Genentech, Bayer, GlaxoSmithKline, Nanocarrier, Vegenics, Celgene, Northwest Biotherapeutics, Berghealth, Incyte, Fujifilm, D3, Pfizer, Multivir, Amgen, Abbvie, Alfa‐sigma, Agensys, Boston Biomedical, Idera Pharma, Inhibrx, Exelixis, Blueprint Medicines, Altum, Dragonfly Therapeutics, Takeda, National Comprehensive Cancer Network, NCI‐CTEP, University of Texas MD Anderson Cancer Center, Turning Point Therapeutics, Boston Pharmaceuticals, Novartis, Pharmamar, Medimmune; VS is also an advisory board/consultant position with Helsinn, Incyte, QED Pharma, Daiichi‐Sankyo, Signant Health, Novartis, Relay therapeutics, Roche, Medimmune; travel funds from Pharmamar, Incyte, ASCO, ESMO; other support from Medscape; all outside the submitted work.
